# Nicotinic Receptor Activity Alters Synaptic Plasticity

**DOI:** 10.1100/tsw.2001.74

**Published:** 2001-08-17

**Authors:** John A. Dani

**Affiliations:** Division of Neurology, Baylor College of Medicine, Houston, TX 77030-3498, USA

**Keywords:** nicotine, addiction, dependence, Alzheimer’s disease, LTP, hippocampus, synapse, potentiation, depression

## Abstract

Studies using specific agonists, antagonists, and lesions have shown that nicotinic cholinergic systems participate in attention, learning, and memory[1,2]. The nicotinic manipulations usually have the greatest influence on difficult tasks or on cognitively impaired subjects[2]. For example, Alzheimer's disease is characterized by a loss of cholinergic projections and nicotinic acetylcholine receptors (nAChRs) in the cortex and hippocampus[3]. Nicotine skin patches can improve learning rates and attention in Alzheimer's patients[4].

Nicotine also is the main addictive component of tobacco, which is estimated to cause 4 million deaths per year worldwide. In developed countries smoking is estimated to be the largest single cause of premature death[5]. The addiction process also entails a kind of learning as the addictive drug reinforces salient and extraneous factors that are consistently a part of the drug experience. Associative learning arises as the addictive drug (e.g., nicotine) initiates, influences, and alters normal cellular mechanisms[6,7]. In particular, forms of synaptic plasticity, such as short-term potentiation (STP) and long-term potentiation (LTP), are cellular mechanisms that modify the communication between synapses and participate during learning and memory.

An important structure for learning and memory is the hippocampus, which has abundant cholinergic innervation and dense expression of nAChRs[8]. LTP has been extensively studied in the CA1 region of the hippocampus, where the NMDA subtype of glutamate receptors initiates a calcium-dependent intracellular process that produces the synaptic potentiation. Inhibitory GABAergic interneurons can modulate the induction of synaptic plasticity by inhibiting the CA1 pyramidal neurons[9]. In a recent study of mouse hippocampal slices[10], my co-workers and I showed that nAChR activity can enhance or depress synaptic plasticity, and the form of the modulation depends on the location and timing of the nAChR activity.

Synaptic potentiation of excitatory glutamate synapses usually arises from the coincidence of presynaptic glutamate release and postsynaptic excitation consequent to that release. At glutamate synapses, $Mg^{2+}$ causes a voltage-dependent ion-channel block of NMDA glutamate receptors. If the postsynaptic depolarization is sufficient, $Mg^{2+}$ is driven out of the NMDA receptors which are then able to conduct the $Ca^{2+}$ current that initiates synaptic potentiation. While studying the glutamatergic Schaffer collateral synapses onto hippocampal CA1 pyramidal neurons, we found that nAChR activity enhances the induction of synaptic potentiation in two main ways[10]. First, there are nAChRs located on some Schaffer collateral presynaptic terminals, and activation of those nAChRs enhances glutamate release. In the presence of presynaptic nAChR activity, a given stimulation of the Schaffer collateral pathway causes greater glutamate release. Second, there are nAChRs located postsynaptically on some CA1 pyramidal neurons, and activation of those nAChRs excites the postsynaptic neuron. The postsynaptic nAChRs mediate a depolarizing current partially carried by calcium. The depolarization helps to relieve the $Mg^{2+}$ block of the NMDA receptors, and the nAChR-mediated $Ca^{2+}$ influx adds onto that mediated by the NMDA receptors. Both the depolarization and $Ca^{2+}$ influx boost the postsynaptic glutamate excitation and increase the probability of synaptic potentiation. The timing of the nAChR activity also is important. When the nAChR activity enhances the coincidence between presynaptic release and consequent postsynaptic response, synaptic potentiation is boosted. If the nAChR activity occurred such that it caused the postsynaptic response before presynaptic glutamate release, then synaptic depression or no change is more likely than synaptic potentiation.

Nicotinic AChR activity was also found to act indirectly to diminish or prevent the induction of synaptic potentiation[10]. As seen in rat hippocampus[1,9,11], nAChRs are much more highly expressed on mouse inhibitory GABAergic interneurons than on pyramidal neurons[10]. Sufficient nAChR activity on a GABAergic interneuron causes it to fire action potentials and produce inhibitory $GABA_A$ receptor synaptic currents onto postsynaptically connected pyramidal neurons[9,10]. By inhibiting the pyramidal neuron, the ACh-induced GABAergic activity prevented the postsynaptic glutamatergic depolarization that is required for the induction of synaptic potentiation. The timing of the nAChR activity was again important. The nAChR-induced GABAergic inhibition had to be timed to arrive just before and during the stimulation used to induce synaptic potentiation.

This study helps to provide a cellular explanation for the nicotinic cholinergic influences on attention, learning, and memory that were known from behavioral results[10]. Depending on the distribution and the timing of nAChR activity, synaptic events can be modified in different ways. Presynaptic nAChRs can increase the probability of neurotransmitter release, increasing the fidelity of synaptic transmission. Postsynaptic nAChRs can increase the depolarization and calcium signal associated with successful transmission. On the other hand, nicotinic activity can potently act upon GABAergic interneuron activity, which regulates the excitability of circuits[9]. The location of nAChR activity and the moment-by-moment change in that activity can tip the balance in favor or against the induction of synaptic plasticity. Because of the wide distribution of nAChRs and broad cholinergic innervation throughout the brain, nicotinic mechanisms like those described here participate in learning, memory, and attention, as well as sleep cycle disorders, analgesia, Tourette's syndrome, epilepsy, and Alzheimer's disease[3]. Furthermore, during the addiction process, nicotine from tobacco misdirects the mechanisms that usually subserve learning and memory[6,7]. Although there is still much to learn, we are beginning to understand the diverse mechanistic contributions of neuronal nAChRs.
